# Effects of disliked music on psychophysiology

**DOI:** 10.1038/s41598-023-46963-7

**Published:** 2023-11-24

**Authors:** Julia Merrill, Taren-Ida Ackermann, Anna Czepiel

**Affiliations:** 1 Max Planck Institute for Empirical Aesthetics, Grüneburgweg 14, 60322 Frankfurt am Main, Germany; 2 Institute of Music, University of Kassel, Kassel, Germany

**Keywords:** Psychology, Human behaviour

## Abstract

While previous research has shown the positive effects of music listening in response to one’s favorite music, the negative effects of one’s most disliked music have not gained much attention. In the current study, participants listened to three self-selected disliked musical pieces which evoked highly unpleasant feelings. As a contrast, three musical pieces were individually selected for each participant based on neutral liking ratings they provided to other participants’ disliked music. During music listening, real-time ratings of subjective (dis)pleasure and simultaneous recordings of peripheral measures were obtained. Results showed that compared to neutral music, listening to disliked music evoked physiological reactions reflecting higher arousal (heart rate, skin conductance response, body temperature), disgust (levator labii muscle), anger (corrugator supercilii muscle), distress and grimacing (zygomaticus major muscle). The differences between conditions were most prominent during “very unpleasant” real-time ratings, showing peak responses for the disliked music. Hence, disliked music has a strenuous effect, as shown in strong physiological arousal responses and facial expression, reflecting the listener’s attitude toward the music.

## Introduction

Many studies have demonstrated the positive effects of music, elucidating why people enjoy music listening^1–6^. Indeed, listeners experience strong reactions to liked music, e.g., chills or ‘thrills,’ charactised by extremely pleasant responses to music^7–9^. However, people can also experience strong reactions to disliked music^10–13^. Such reactions can be so strong that music is even used in psychological warfare (for example, very loud techno or heavy metal music)^14^. Only recently have studies evaluated the rationales and functions of disliked music and thereby showing the evaluative diversity and complexity with which people approach, think, and talk about music.

A key reason for disliking music is the evoked feeling of displeasure. Such displeasure ranges from slight feelings of unrest to strong physical reactions such as disgust and pain, leading people to leave the room or even break off social contact^12,13,15^. Conversely, the feeling of pleasure was seen as evidence for the positive effects of music, which was measured with responses of the autonomic nervous system (ANS). Resulting high arousal responses were interpreted as reflecting the reward humans experience during music listening^4,16^. But, if the physiological responses to one’s liked music are interpreted as reflecting pleasure and reward and is used to give answers as to why people like to listen to music, the physiological responses to one’s disliked music should provide insights as to why people do not enjoy listening to their disliked music. In the current study, psychophysiological reactions to one’s personally disliked music were compared to musical pieces that were considered neutral. Responses from the ANS should reveal the distress und displeasure people can feel when listening to their own disliked music.

Traditionally, pleasure and displeasure in response to music have often been investigated with pre-evaluated musical stimuli, that is, music that was pre-rated on arousal and valence or (basic) emotions^17–23,23,24^. The idea of the current study differs from this approach as music is not per se positive or negative or evokes a high or low arousal response, but is liked or disliked because of the participant’s personal attitude or experience. Interestingly, studies investigating musical reward have followed this exact approach where the physiology reflects responses in a listener that are beyond the ascribed emotion of the music, i.e., preference. Some of these studies have investigated musical chills using a unique study design, focusing on chills as a subjective experience of liking the music. Participants were invited to bring their favorite, chill-evoking music. Importantly, the acoustic features were controlled for by matching each participant with a second participant who rated the same piece of music as eliciting neutral responses. This would show that responses to the music are driven by the personal attitude toward the music, regardless of the acoustic signal. The findings showed that felt pleasure and chills correspond to heightened arousal such as increased heart rate (HR)^4,25,26^, and skin conductance response (SCR)^4,17,23,27,28^, as supported by neuroimaging studies showing that pleasurable music activates typical reward structures and networks^25,29^, as well as increasing dopamine release^30^. A recent study even showed that a dopamine precursor (levodopa) increased pleasurable experiences with music. Using SCR as an index of music-evoked pleasure, SCR increased with the dopamine precursor but decreased under the dopamine antagonist^16^.

Notably, participants in these chill studies were selected because of their personal attitude toward the music, that is one’s favorite music that reliably evoked highly pleasurable moments such as chills. When investigating disliked music, the specific attitude would therefore need to be controlled, too, particularly because several reasons were shown to be behind musical dislikes^13^. Here, three main groups of value judgments have been determined. Firstly, music-related reasons refer to the melody, the harmony, or rhythm of the music, which are judged as not fitting certain musical expectations (e.g., not being melodic enough, or being too disharmonic). Secondly, personal reasons refer to the music not fitting own beliefs or values, or not meeting emotional expectations such as evoking displeasure or having no effect on the listener (e.g., “the music does not do anything to me”). Thirdly, social reasons refer to a mismatch with the people who listen to that music, or the music not being part of one’s in-group (family or friends). To be in line with the aforementioned chill studies, participants need to be selected who dislike the music because it leads to highly arousing, unpleasant feelings—and not other reasons such as the music having no effect.

When experiencing displeasure, high arousal moments should be expected because of anger, annoyance, and distress. Indeed, arousing (e.g., happy or angry) music evokes increased SCR, HR, and respiration rate (RR), compared to low arousal (e.g., sad or relaxing) music^18,19,22,23,31^. SCR increased with less preferred music^32^ and fearful music^18^, HR decreased in response to negative/unpleasant stimuli^33–35^, and body temperature decreased with fearful music^19^. Still, studies show deviating results depending on the experimental conditions^21,36^.

While the above measures indicate arousal, measurements of facial electromyography (EMG) were used to indicate valence. Typically, activations of the zygomaticus major and corrugator supercilii muscles are measured, which become activated when one smiles or frowns, respectively. Thus, zygomaticus activation has been associated with positive valence, while corrugator activation has been associated with negative valence in response to visual^37^ and certain auditory stimuli^20,22,38–40^. However, there is growing contradictory evidence suggesting that zygomaticus activity does not reflect felt positive valence alone. Dissonant music (rated as unpleasant/lower in liking) was found to increase zygomaticus activity compared to consonant music (rated as pleasant/liked) potentially because in these cases, such activation may represent tension and grimacing because of distress, rather than smiling^41–44^. It also seems that one facial muscle is active in the expression of more than one emotion, most in combination with other muscles, e.g., the activity of the levator labii superioris alaeque nasi muscle (short: levator labii; activated during nose wrinkling) increased for both disgust and happiness, and the zygomaticus activity increased for both fear and happiness^45^. Hence, the question remains on how EMG will reflect personal attitudes toward music.

Interestingly, no study has asked participants to bring their disliked music into the laboratory to research these strong negative attitudes. Conversely to chill-evoking liked music, investigating the physiological responses to truly disliked music will provide us with evidence of why participants avoid their disliked music, i.e., if the music can evoke these strong negative effects that participants report and use to explain their dislikes^13^. By following the study design and selected physiological measures of the chill studies presented above^4^, in the current study, participants listened to three self-selected disliked musical pieces and three neutral pieces from the other participants. During music listening, real-time ratings of subjective (dis)pleasure and simultaneous recordings of the ANS were obtained in addition to facial muscles such as levator labii (nose wrinkling), corrugator supercilii (frowning), and zygomaticus major (smiling) muscles. The implementation of real-time ratings would facilitate the differentiation of moment-to-moment responses from overall attitudes. Specifically, it would allow for the identification of indicators of displeasure that may manifest independently of the neutral or disliked musical pieces.

We expected that disliked music, and at real-time moments of strong displeasure, would evoke typical arousal responses (increased SCR, RR, HR, and body temperature) compared to neutral music. Considering the traditional view of EMG, we might hypothesize that EMG for smiling muscle would decrease, while EMG for frowning and nose wrinkling muscles would increase during disliked music. However, with increasing evidence suggesting that EMG does not clearly map onto positive/negative valence, we hypothesize that EMG muscle activity would change in the dislike condition, but did not predict a certain direction.

Participants were selected based on a specific attitude toward their disliked music to optimize strong responses to music. As high arousal displeasure was a prerequisite for participation, it was hypothesized that participants might experience anger and stress more frequently and severely than the normal population. Additionally, personality traits and aspects of musical sophistication might reveal a different emotional or cognitive engagement with music.

## Methods

### Ethics statement

All experimental procedures were approved by the Ethics Council of the Max Planck Society (No 2702-12) and were undertaken with written informed consent of each participant. All research was performed in accordance with the Declaration of Helsinki.

### Participants

Forty-one participants (21 female) took part in the psychophysiology study, with a mean age of 43.41 years (SD = 16.26, range 20–72). For the highest school education level, 37 completed the A-levels (German “Abitur”), three participants completed high school after 10 years, and one chose not applicable. As the highest educational qualification, 19 had a postgraduate degree, five a Ph.D. or M.D., three a Bachelor’s degree, three were university students, eight had a professional qualification, one had no professional qualification, and two chose not applicable.

**Figure 1:**
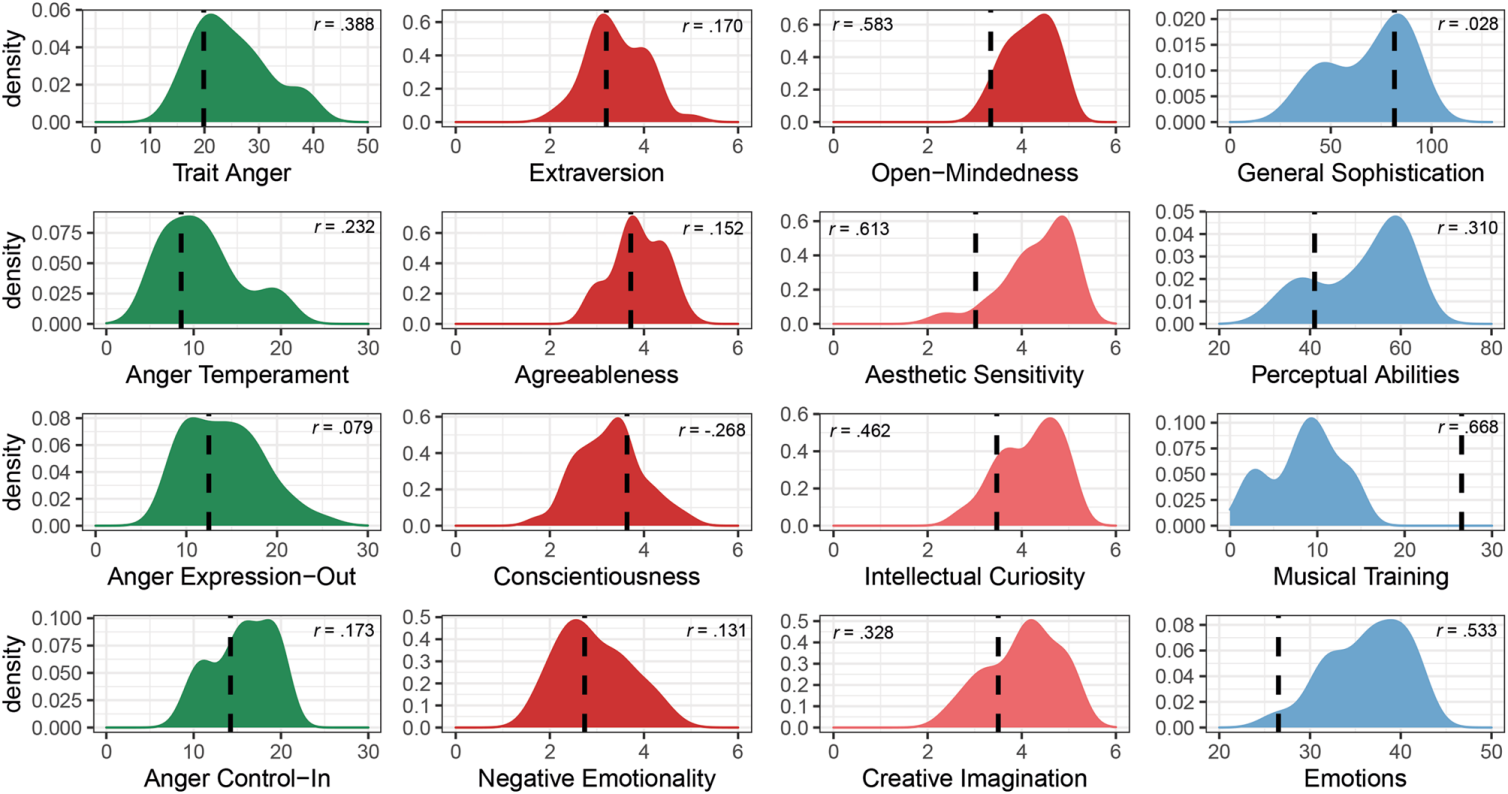
Character traits. Density plots of Trait Anger (green; sum score), personality traits (red; mean rating) and facets of Open-Mindedness (light red), Musical Sophistication (blue; sum score) General and factors. Dashed lines show the mean of the normative groups.

To draw conclusions about specificities of the sample, the collected trait measures were compared to normative groups from the original publications by calculating the effect size *r*, with a small effect size being *r* = 0.1, medium *r* = 0.3, and large* r* = 0.5^46^. Participants scored higher in Trait Anger than the normative group (STAXI-2^47^, *r* = 0.388; Fig. 1), i.e., they show the tendency to perceive a wider range of situations as disturbing or frustrating and to react in such situations by increasing their level of anger (full reports in Supplementary Table S1). Concerning personality traits (BFI-2^48^), participants were higher in Open-Mindedness (*r* = 0.583), particularly in Aesthetic Sensitivity (*r* = 0.613). Regarding general musical sophistication, participants did not score differently than the German normative group (Gold-MSI^49^, *r* = − 0.028). However, they scored much lower in musical training (*r* = − 0.668) but higher in emotions (*r* = 0.533), and perceptual abilities (*r* = 0.310). Differences between the groups in perceived stress reactivity (PSRS^50^) were small, e.g., only a small effect for the scale of perceived stress reactivity to failure was found (*r* = 0.238), and no difference in the need for cognitive closure scale (16-NCCs^51^; *r* = 0.018).

### Participant selection

The participants were selected based on an online survey and the matching between participants was done in an in-person session. The online survey was conducted with 529 participants (EFS/unipark) in which interested participants named three musical pieces that annoy, stress out, or similar. Each named piece was rated on how much it was disliked on a 5-point Likert scale from “very low dislike” to “very strong dislike.” Then, a list of 13 rationales for disliking the piece was presented^13^, and rated on a 5-point Likert scale from “strongly disagree” to “strongly agree.” The five reasons related to displeasure should strongly apply (e.g., “evoking unpleasant feelings” and “feeling stressed”), the others should not apply as they do not reflect high arousal (i.e., “the music does not affect the mood, makes one sad, or one is not moved by it”) and were not of interest to the current research aim (the exact selection criteria are described in Supplementary Materials). Finally, the participants were asked if they disliked the piece only because of the association of a certain negative event (yes/no).

The online survey ended after 88 people were found who stated high displeasure to be the reason for disliking their chosen music, which was in accordance with the chill studies which only included participants who reported reliable chills in response to their liked music. Note that negatively associated memories with the song led to exclusion. Altogether, 88 participants (48 female) were invited to the first in-person session, which was attended by 47 participants (25 female), giving liking ratings on 30-s excerpts of each song provided by the other participants. Once all participants had completed this session, for each disliked song by one participant, a match by another participant who rated the song in the middle of the rating scale was determined^4^. Only for one song, no match could be found (the exact matching procedure can be found in Supplementary Materials). This ensured that in the main session, each song was listened to by two participants, of which one listened to it in the neutral condition and the other in the dislike condition.

### Procedure

Forty-one participants returned to the main session. After written informed consent, participants were prepared for the physiological measures (for detailed information on the apparatus and the exact procedure see Supplementary Materials).

The procedure started with filling out questionnaires evaluating the participants’ current mood (PANAVA-KS^52^, 7-point scale, bipolar items) and state anger (STAXI-2, part 1). Then, participants listened to six three-minute-long pieces: the three self-selected disliked pieces and the three pieces by other participants with neutral ratings, evaluated in session #1. Disliked and neutral pieces were counterbalanced: half of the participants listened to the three disliked pieces at first, and half of them started with the neutral pieces. The order of pieces within these blocks was randomized. A custom-made user interface^53^ was employed to program the experimental scenario. The study was presented with the software Presentation (Neurobehavioral Systems Inc.). After each piece, questions about the liking from “strongly dislike” (1) to “like very much” (7) were acquired to check again the general attitude toward the music, and the pleasantness from “very unpleasant” (1) to “very pleasant” (7) to check the grade of displeasure that was reported in the selection process as a reason for the dislike. Then, the PANAVA-KS, as well as the music-evoked feelings during listening were acquired (retrospectively) with a subset of items from the selection process on a 5-point Likert scale from “strongly disagree” (1) to “strongly agree” (5), again to reaffirm the previous ratings: “It triggered unpleasant feelings, it put me in bad mood, it was physically uncomfortable, I found it boring, it had no effect on my mood, it made me sad, it touched me.” This was repeated for each piece. After the first and the second block (neutral or disliked), the STAXI-2 part 1, was assessed again. The music was presented in blocks of disliked and neutral music because of cortisol probes that were taken additionally and cortisol has a delay of several minutes in response to stress. Note, the cortisol probes were analyzed by an external laboratory and did not contain any cortisol, which was probably the result of a measurement failure, thus is not reported.

Additionally, while participants listened to the music, they were asked to continuously provide real-time ratings on the felt (un)pleasantness with a button press from (1) pleasant, (2) neutral, (3) unpleasant, to (4) very unpleasant. This non-symmetrical rating scale was chosen because no ‘very pleasant’ ratings could be expected in this study. The ratings were assessed with the hand not occupied with measurements. The buttons were pressed on a keyboard and participants were instructed to keep the finger on the button all the time and change as fast as possible between the buttons. The questionnaires were filled out with a computer mouse, all with the right hand. The preparation took about 20 min and the measuring took about 45 min.

After participants had taken off the measures, they filled out further questionnaires on a laptop computer. This included the questionnaires STAXI-2 parts 2 and 3 on trait anger, BFI-2, 16-NCCS, Gold-MSI, and PSRS, in this order. This part took about 30 min. Participants received monetary compensation of 30 euros for each session.

### Analysis

#### Preprocessing

Preprocessing of physiological responses was conducted in MatLab 2019b (The MathWorks) (for details see Supplementary Materials). To observe within-excerpts differences, physiology was cut and categorized depending on the button presses. Post-hoc triggers were inserted into the data at timestamps of button presses and the physiology was taken from three seconds before and nine seconds after this time point following typical epoch times of previous studies^34,41^. These were transformed into four 3-s time windows:^4^ pre-button press, first, second, and third time window.

Note that there was an unequal number of observations between the button presses in the conditions with particularly fewer numbers of neutral and pleasant ratings in the dislike condition and fewer numbers of very unpleasant ratings in the neutral condition: Neutral rating: dislike *n* = 4903, neutral *n* = 11,138; pleasant rating: dislike *n* = 1097, neutral *n* = 4959; unpleasant rating: dislike *n* = 9034, neutral *n* = 8642; very unpleasant rating: dislike *n* = 8482, neutral *n* = 2340.

#### Statistical analysis

All statistical analyses were done using R version 4.1.1. For each z-transformed psychophysiological measure, one linear mixed-effects model was fitted with the fixed effects and their interaction terms of time window (four time windows: pre, 1st, 2nd, 3rd), condition (two levels: neutral and disliked music), rating (four levels: neutral, pleasant, unpleasant, very unpleasant), and the random intercept of participant. As the model would not converge with maximal random effects structure (that is, random intercept and random slopes for participants), a reduced random-effects model (that is, with random intercept of participant only) was used to optimally fit the data^54^. Nonetheless, we note a potential limitation that the results have a higher risk of Type I errors (false positives) due to the simplified random effects (and inflated degrees of freedom). Reported are the main effect of condition and the two-way interaction (IA) of condition × rating from an ANOVA with Satterthwaite’s method and their Tukey pairwise comparisons.

Behavioral differences between conditions (neutral, dislike) in liking and pleasantness ratings, state anger, mood, and music-evoked feelings were investigated with linear mixed-effects models with condition as the fixed effect and participant as random intercept for each variable. As for the physiology, the ANOVA results are reported. For the music-evoked feelings, a factor analysis with oblimin rotation was performed to create latent variables for the models. Using a parallel analysis, three factors were identified, the first including ‘unpleasant feelings’ (it triggered unpleasant feelings, it put me in bad mood, it was physically uncomfortable), the second ‘boring/no effect’ (I found it boring, it had no effect on my mood), and the third ‘sad/touched’ (it made me sad, it touched me; see Supplementary Table S2).

## Results

### Psychophysiology

**Figure 2:**
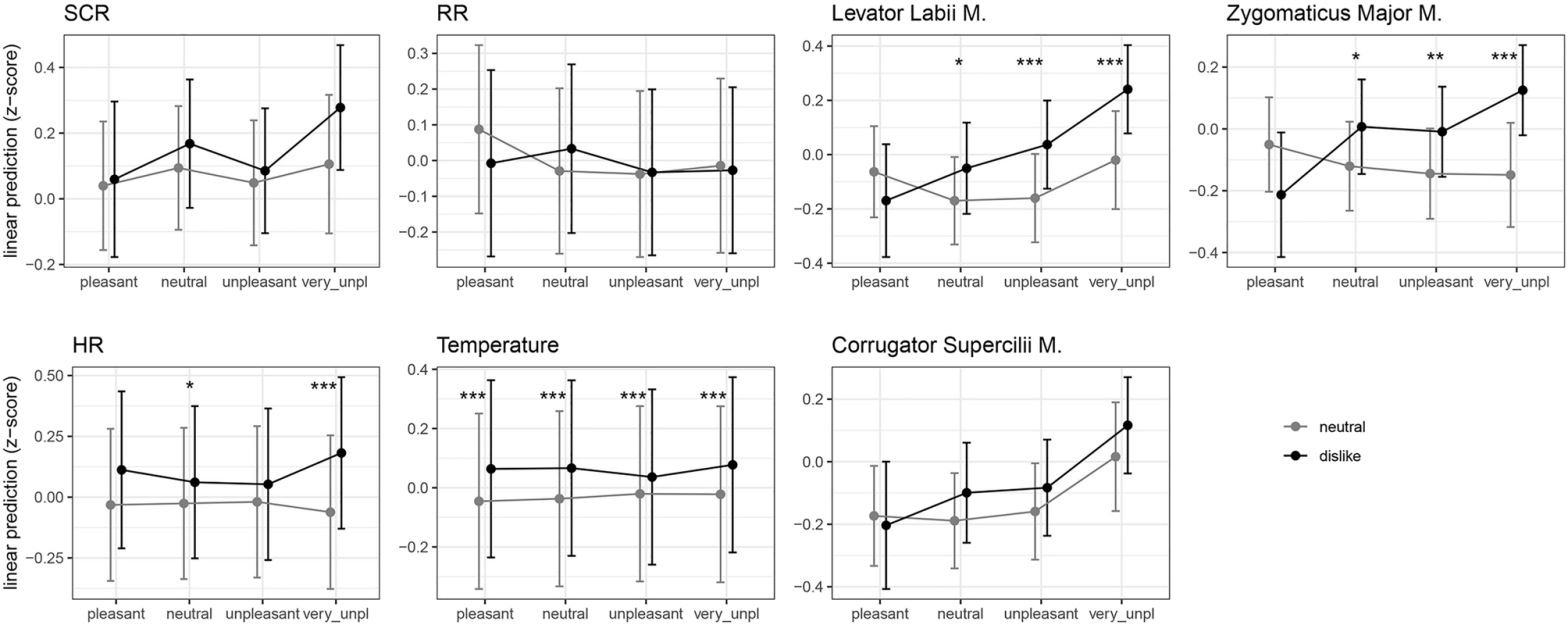
Interaction plot of estimated marginal means (with confidence intervals) based on the fitted models with condition and rating. Significant pairwise comparisons are indicated with asterisks (*** *p* < 0.001, ** *p* < 0.01, * *p* < 0.05).

**Figure 3:**
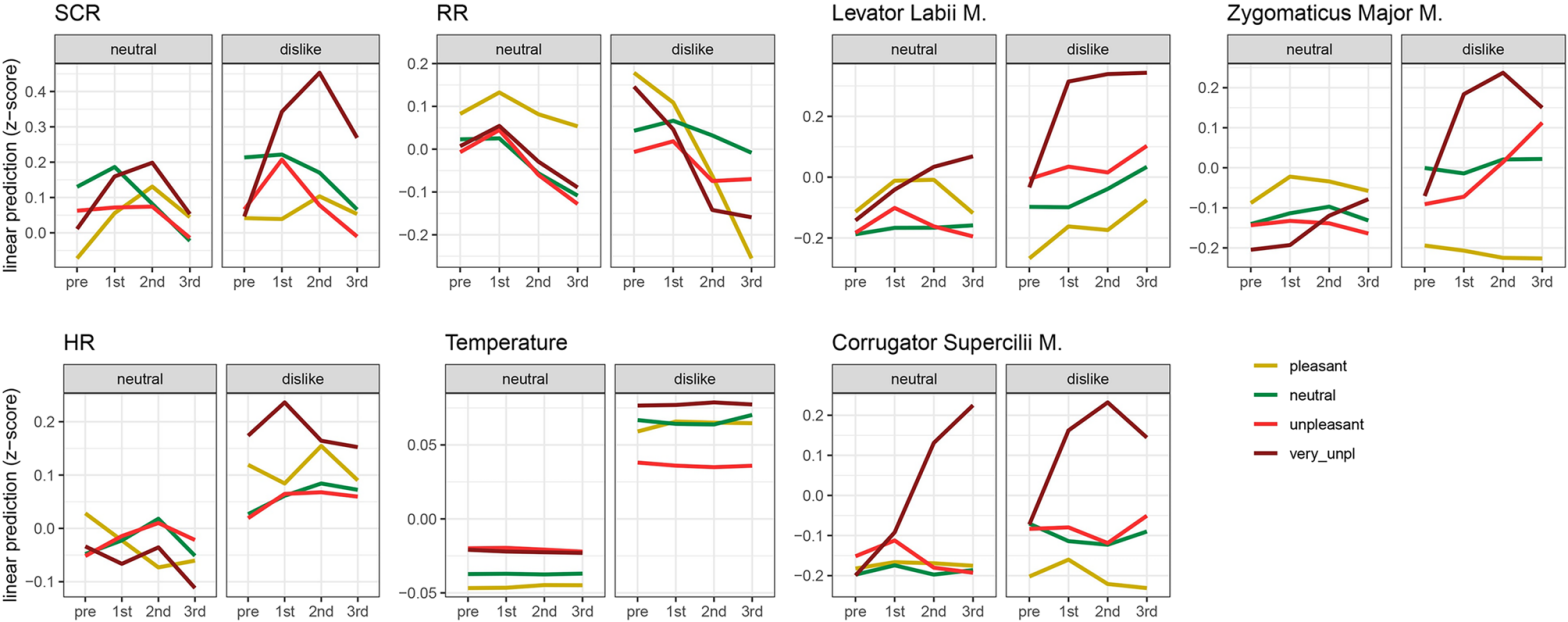
Interaction plot of estimated marginal means based on the fitted models with condition, rating, and time windows.

The response to the disliked music is higher overall compared to the neutral music showing that a strong negative attitude toward one’s disliked music leads to an increase in physiological arousal and facial expression. The data revealed a strong association between the subjective ratings of displeasure and the psychophysiological measures and facial muscle activity. Figure 2 demonstrates increases in SCR, HR, and body temperature, as well as EMG response, as participants report experiencing more displeasure with the musical pieces. Figure 3 demonstrates descriptively the event-related response of the measures related to the real-time ratings and the conditions of disliked vs. neutral music.

#### Main effect of condition (dislike vs. neutral)

A significant main effect of condition revealed a higher response for the dislike than the neutral condition in SCR (*F*(1, 6423) = 6.2, *p* = 0.013, *R*^*2*^(m) = 0.012, *R*^*2*^(c) = 0.298), HR (*F*(1, 5245) = 51.9, *p* < 0.001, *R*^*2*^(m) = 0.006, *R*^*2*^(c) = 0.782), temperature (*F*(1, 7091) = 100.2, *p* < 0.001, *R*^*2*^(m) = 0.002, *R*^*2*^(c) = 0.922), as well as levator labii (*F*(1, 7110) = 19.0, *p* < 0.001, *R*^*2*^(m) = 0.028, *R*^*2*^(c) = 0.294), corrugator supercilii (*F*(1, 7113) = 4.5, *p* < 0.034, *R*^*2*^(m) = 0.016, *R*^*2*^(c) = 0.251), and zygomaticus major muscles (*F*(1, 7117) = 10.4, *p* = 0.001, *R*^*2*^(m) = 0.013, *R*^*2*^(c) = 0.212) (all main effects and interactions in Supplementary Table S3). Only in RR, the main effect condition did not become significant (*p* = 0.70). Hence, physiological arousal measures as well as muscle activity related to frowning (corrugator), nose wrinkling (levator labii), and smiling (zygomaticus) were significantly higher when listening to disliked music compared to neutral music.

#### Interaction effects of event-related responses and conditions

Since music is a dynamic stimulus, we further investigated differences in the real-time ratings of (un)pleasantness between dislike and neutral conditions. Therefore, the two-way IA of condition × rating and post hoc pairwise comparisons of the ratings between conditions were calculated.

In SCR, the IA of condition × rating did not become significant (*p* = 0.260), i.e., the response during the real-time ratings showed a similar pattern in both conditions (Fig. 2; all statistics in Supplementary Table S4). However, the time course of the real-time ratings in Fig. 3 shows that for most ratings in both conditions, there was a typical rise and fall in SCR, but that this peak was higher and steeper in the very unpleasant rating in the dislike condition (but not significantly different from the neutral condition).

For HR, there was a significant IA between condition and ratings (*F*(3, 5241) = 5.8, *p* < 0.001). Pairwise comparisons revealed that HR was higher during very unpleasant and neutral ratings in the dislike condition than in the neutral condition (Fig. 2). Figure 3 shows a strong increase after the button press for the very unpleasant ratings in the dislike condition, but there was also a visible increase for the pleasant ratings in the 2nd time window.

RR did not show a significant IA (*p* = 0.20), hence, the response during ratings showed a similar pattern for both conditions (Fig. 2). RR is the only measure that showed a decrease after the button press, which was particularly strong in the dislike condition for the very unpleasant and pleasant ratings in the dislike condition (Fig. 3).

Temperature also revealed a significant IA (*F*(3, 7090) = 3.2, *p* = 0.02) and the response was higher for all ratings during the dislike condition than the neutral condition as shown by pairwise comparisons (Fig. 2). The temperature was the only measure that did not show an event-related response to the button press as no peaks can be observed over time (Fig. 3).

The levator labii muscle revealed a significant IA (*F*(3, 7099) = 6.0, *p* < 0.001), with a significant increase for the very unpleasant, unpleasant, and (less strongly) neutral ratings in the dislike condition (Fig. 2). Figure 3 shows that in the dislike condition, the levator labii had a significant peak larger in amplitude in the very unpleasant compared to all other ratings.

Zygomaticus muscle patterns were similar to the levator labii, with a significant IA (*F*(3, 7103) = 6.4, *p* < 0.001), and significant pairwise comparison showing a stronger increase for the very unpleasant, unpleasant, and (less strongly) neutral ratings during the dislike than the neutral condition (Fig. 2). Figure 3 shows that in the dislike condition, zygomaticus activity increased after the button press for very unpleasant and unpleasant ratings.

For the corrugator muscle, the IA did not became significant (*p* = 0.538) (Fig. 2). Figure 3 shows that corrugator activity increased to a peak for the very unpleasant rating in both the dislike and the neutral conditions.

### Behavioral response to the musical pieces

**Figure 4:**
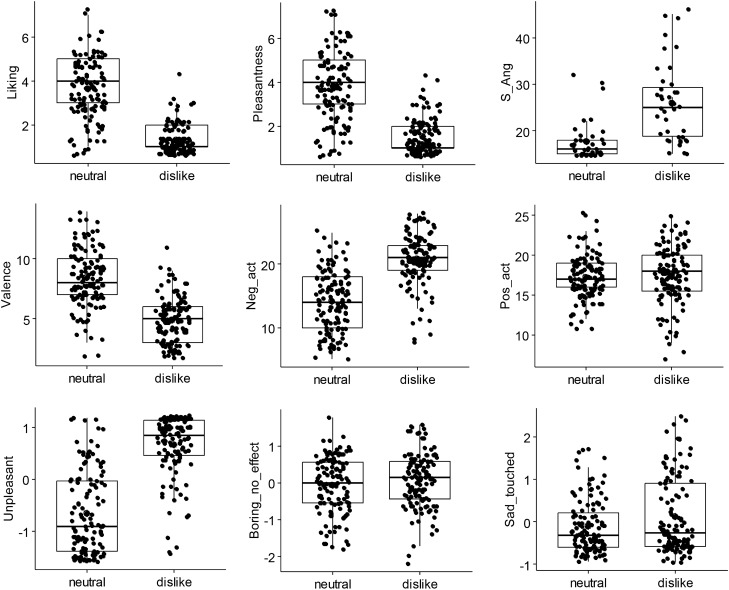
Boxplots of behavioral ratings for neutral and disliked music with liking and pleasantness ratings, state anger (sum score; upper row), mood (sum scores; middle row), and latent variables of music-evoked feelings (factor scores; bottom row).

After the pieces, participants rated the music regarding liking, pleasantness, state anger, mood, and music-evoked feelings. Significant main effects showed that the disliked music was rated lower in liking (*F*(1, 204) = 312.45, *p* < 0.001, *R*^*2*^ = 0.536), and pleasantness (*F*(1, 204) = 247.24, *p* < 0.001, *R*^*2*^ = 0.451), but higher in state anger than neutral music (S-Ang; *F*(1, 39.87) = 40.839, *p* < 0.001, *R*^*2*^ = 0.282; Fig. 4; descriptive statistics in Supplementary Table S5). The subscales of verbally expressed anger (S-Ang-V; *F*(1, 40.09) = 34.423, *p* < 0.001, *R*^*2*^ = 0.273), feeling angry (S-Ang-F; *F*(1, 39.32) = 42.788, *p* < 0.001, *R*^*2*^ = 0.260), and physically expressed anger (S-Ang-P; *F*(1, 40.12) = 14.472, *p* < 0.001, *R*^*2*^ = 0.132; not depicted) were all higher for disliked than neutral music. That means, during listening to their disliked music, participants report experiencing higher emotional states of tension, disturbance, irritation, or rage/anger than during the neutral condition. It is of note, though, that a floor effect could be seen for the S-Ang ratings and its subscales, i.e., participants scored low on the 5-point scale (S-Ang: dislike: *M* = 1.73, neutral: *M* = 1.18).

Concerning the mood changes (PANAVA-KS), negative activation (*F*(1, 204) = 229.58, *p* < 0.001, *R*^*2*^ = 0.372) and negative valence (*F*(1, 204) = 245.84 *p* < 0.001, *R*^*2*^ = 0.408) were also higher for disliked music. Interestingly, this effect was not symmetrical in that no significant effect of positive activation was found (*p* = 0.512).

From the music-evoked feelings, ‘unpleasant feelings’ were higher for disliked music (*F*(1, 204) = 294.41, *p* < 0.001, *R*^*2*^ = 0.469). While ‘boring/no effect’ (*p* = 0.089) did not become significant, ‘sad/touched’ (*F*(1, 204) = 10.947, *p* < 0.01, *R*^*2*^ = 0.023) became significant revealing a small effect size. Taken together, the results show that the assumptions were met that disliked music led to self-reported high negative arousal.

## Discussion

From mood management to cognitive enhancement, music has always been praised for its positive effects. However, these effects are dependent on the listener’s attitude; not all music has the same effect on all listeners. The fact that listening to one’s favorite music can evoke chills that are visible in physiological responses was used as a rationale to show the positive, rewarding effects of (one’s most liked) music. The current study investigated for the first time the physiological responses to one’s disliked music, revealing the negative effects caused by a specific attitude toward music.

The results overall show that listening to personally disliked music—compared to matched neutral music—leads to negative behavioral and higher peripheral responses. As expected, self-reported liking, pleasantness, and positive valence were lower, while unpleasant feelings and state anger were higher for disliked than neutral music. Physiological reactions show an increase in facial muscle activity as well as higher arousal, namely significant increases in HR, SCR, and body temperature, but not in RR. This is in line with other studies showing heightened arousal during music-evoked emotions^18,19^ and listening to music with low vs. high liking ratings in particular, where SCR was an indicator for unpleasant music in comparison with neutral music^40^ and with liked music, while RR was not^32^. Notably, these arousal measures were seen to respond to intensely pleasurable, chill-inducing music as well^4^, but were interpreted to reflect the rewarding and overall positive effects of music listening. Albeit listening to one’s disliked music leads to similar responses, the rationale behind it differs. The current findings show that the reported feeling of displeasure in response to one’s disliked music can lead to measurable physiological responses, reflecting the distress that music can evoke in people with a particular attitude toward the music. In the neutral condition, where participants do not have such a strong attitude, they do not show a corresponding response.

Besides higher arousal responses, facial muscle activity was also higher for disliked than neutral music. The levator labii (‘nose wrinkling’) muscle showed the strongest effect in the current study, supporting the idea that an increased activity for disliked music might reflect negative feelings such as disgust^24,45^. The corrugator muscle, which was in previous studies found to reflect negative emotions in music^19,24,40,55^ was also higher for disliked music, but was in both conditions (disliked and neutral) higher during the very unpleasant moments and equally low for the pleasant moments. Therefore, the corrugator seems to be a consistent indicator of very unpleasant feelings, albeit less dependent on the condition and hence, the general attitude of the listener.

While higher activity of the corrugator was to be expected for disliked music because it is involved in negative expression such as anger^45,56^, a higher zygomaticus activity was not necessarily expected because of its involvement in music with a happy/joyful expression^19,40,55^. Nonetheless, previous studies have shown the activation of the zygomaticus in dissonant music and less-liked music and have been interpreted as revealing distress and/or grimacing in response to music^41–43^. Notably, when inspecting the differences in real-time ratings between the conditions, the zygomaticus activity seems to depend on the attitude toward music as well as the moment-to-moment responses. In the neutral condition, zygomaticus activity was highest in pleasant ratings, while in the dislike condition, it was highest in very unpleasant ratings. The response can therefore indicate pleasant as well as unpleasant feelings depending on the general attitude of the listener toward the music, that is, a strong and stable dislike can change the expected response of facial muscles.

In the arousal measures, the contrast between a specific attitude and the moment-to-moment responses was less obvious than for the EMG measures. While SCR showed a systematic event-related response which peaked at about three to four seconds after the button press, this pattern was most prominent upon indicating ‘very unpleasant’ moments (with a stronger visible increase in the dislike condition; Fig. 3). Similarly, HR showed an event-related response that peaked at about three seconds after the button press but more diffusely than SCR. HR mostly increased after the button press, but for some ratings faster than for others, and showed some bimodal curves (accelerations-decelerations) for some ratings. Such patterns are typical of HR responses, where a triphasic deceleration-acceleration-deceleration pattern occurs in orientation responses^34,38,57,58^. Temperature was very stable and did not show a short-term event-related response. Increases and decreases appear on a long-term level as a difference between the conditions can be seen overall. Therefore, differences in body temperature might become visible with longer musical excerpts, which might explain why some studies have not found significant differences in body temperature between conditions or deviating results overall^4,17^. RR did not show a peak and revealed the strongest decrease after the button press of all measures. This pattern is similar between the conditions but with a steeper decline in the dislike condition. Participants slow their breathing or maybe even hold their breath after the change in ratings, particularly during listening to the disliked music (which some of the current participants even reported after the study). Hence, the arousal measures showed overall stronger responses to disliked music which can be explained with the overall higher feeling of displeasure, which was most prominent during moments of very unpleasant feelings. Future studies should investigate further the differences between the measurable effects of a general attitude and momentary evoked responses visible in physiology.

We note that, in all measures, the difference between the neutral and the unpleasant real-time ratings was small and often did not reflect the expected difference of unpleasantness leading to higher responses than neutral. This suggests that the unpleasant ratings as well as the neutral ratings were used as a rating to default to after very unpleasant feelings occurred. This was particularly evident in the EMG measures, where for the dislike condition, neutral and unpleasant ratings were in between the very unpleasant and pleasant ratings. Possibly, the event-related behavior of the measures after the very unpleasant rating marks a peak emotional response, which might be comparable to negative frissons or negative chills^59,60^. Likewise chills occur in response to highly pleasurable moments in liked music^4^, they might also occur during highly unpleasant feelings^61^. Future studies might query participants about their specific chill-like response to disliked music.

As the current sample was selected for their specific attitude toward their music (equally to the participant selection in musical chill studies), it was important to compare a selection of traits with data representing large parts of the population. As expected, participants scored slightly higher in trait anger than the normative group, revealing that they possibly respond to various situations with slightly higher anger expression than the average person, not just during music listening (but note the floor effect of this measure). Further, the self-reported higher emotional engagement with music shows that the participants engage emotionally—both positively and negatively—with music. At the same time, the participants scored higher in Open-Mindedness and particularly high in the facet Aesthetic Sensitivity—a trait associated with higher musical engagement and diverse musical preferences^62–65^. This is a particularly interesting finding as certain phenomena such as the omnivore in musical taste have been discussed^66^, that is people who appreciate a large variety of musical styles and claim to not dislike anything. The current results show that even people high in Openness (and higher level of education) can dislike music so much that listening to it leads to a measurable physiological reaction.

To conclude, emotional arousal in response to liked music has so far been explained with the rewarding aspects of music listening which should answer the question of why humans enjoy listening to music. The current findings have implications for our understanding of the “power of music” as music is capable of evoking strong negative sensations with a measurable physiological effect. Listening to one’s most disliked music results in evoked responses typical of the sympathetic (fight-or-flight) system, thus being related to distress, which has been reported by participants when describing their reactions to their disliked music^12,13^. The current findings align with prior research indicating a relation between increasing levels of felt unpleasantness and heightened physiological arousal. However, the current study further demonstrates that individual attitudes to music can significantly influence these responses. This highlights the importance of evaluating not only the momentary preference for a stimulus, but also the overall attitude of the listener, such as their musical taste and trait aspects, in future investigations.

### Supplementary Information

Supplementary Information 1.

Supplementary Information 2.

## Data Availability

All data is provided as supplementary material.
