# Supplementary material for: Effects of disliked music on psychophysiology
Source: Sci Rep. 2023 Nov 24;13:20641. doi: 10.1038/s41598-023-46963-7 (PMC10674009; doi:10.1038/s41598-023-46963-7)
Supplement: Supplementary file 2 — Supplementary Information 2. [file 41598_2023_46963_MOESM2_ESM.docx]

**The negative power of music: Effects of disliked music on psychophysiology**

Julia Merrill, Taren-Ida Ackermann, & Anna Czepiel

**Supplementary Material**

# Methods

## Participants

Table S1. Comparison of mean ratings (sum scores) of the current sample and the normative groups from the original studies

| **Dimension/facet** | **Mean** | **SD** | **Mean normative** | **SD normative** | **Cohen‘s *d*** | **Effect size *r*** |
| --- | --- | --- | --- | --- | --- | --- |
| **STAXI-2 (Trait Anger)** | | | | | | |
| T_Ang | 25.02 | 6.72 | 19.86 | 5.49 | 0.841 | 0.388 |
| T-Ang_T | 10.93 | 4.36 | 8.57 | 3.18 | 0.618 | 0.295 |
| T-Ang_R | 12.17 | 2.05 | 11.28 | 2.96 | 0.35 | 0.172 |
| AX-O | 14.59 | 4.92 | 12.48 | 3.86 | 0.477 | 0.232 |
| AX-I | 18.12 | 5.09 | 17.36 | 4.46 | 0.159 | 0.079 |
| AC | 31.07 | 5.87 | 28.99 | 5.97 | 0.351 | 0.173 |
| AC-O | 15.46 | 3.42 | 14.78 | 3.36 | 0.201 | 0.1 |
| AC-I | 15.61 | 3.38 | 14.22 | 3.08 | 0.43 | 0.21 |
| **Goldsmiths Musical Sophistication Index** | | | | | | |
| GMSI_active | 33.41 | 9.86 | 32.99 | 9.45 | -0.802 | -0.372 |
| GMSI_perception | 51.78 | 9.58 | 45.84 | 8.62 | 0.182 | 0.091 |
| GMSI_training | 8.51 | 3.84 | 22.85 | 10.62 | -2.111 | -0.726 |
| GMSI_singing | 28.49 | 11.07 | 30.67 | 5.55 | -0.319 | -0.158 |
| GMSI_emotions | 36.29 | 4.19 | 27.55 | 8.87 | 0.352 | 0.173 |
| GMSI_general | 69.32 | 19.49 | 70.41 | 19.94 | -0.611 | -0.292 |
| **Perceived Stress Reactivity Scale** | | | | | | |
| PSRS_total | 25.51 | 8.17 | 23.22 | 7.973 | 0.284 | 0.14 |
| PSRS_AR | 3.15 | 1.93 | 3.22 | 2.198 | -0.034 | -0.017 |
| PSRS_RM | 5.61 | 1.59 | 4.808 | 1.672 | 0.491 | 0.238 |
| PSRS_RsK | 7.10 | 1.90 | 6.393 | 2.17 | 0.348 | 0.171 |
| PSRS_RA | 4.39 | 2.61 | 4.193 | 2.482 | 0.079 | 0.039 |
| PSRS_RsB | 5.27 | 2.37 | 4.6 | 2.388 | 0.282 | 0.139 |
| **Big Five Inventory 2** | | | | | | |
| P_Extraverion | 3.41 | 0.60 | 3.2 | 0.62 | 0.344 | 0.17 |
| P_Agreeableness | 3.88 | 0.53 | 3.72 | 0.51 | 0.308 | 0.152 |
| P_Conscientiousness | 3.28 | 0.68 | 3.64 | 0.61 | -0.557 | -0.268 |
| P_neg_emotionality | 2.93 | 0.74 | 2.74 | 0.7 | 0.264 | 0.131 |
| P_open_mindedness | 4.18 | 0.50 | 3.34 | 0.66 | 1.435 | 0.583 |
| PS_sociability | 3.20 | 0.80 | 3.18 | 0.82 | 0.025 | 0.012 |
| PS_assertiveness | 3.41 | 0.93 | 3.19 | 0.75 | 0.26 | 0.129 |
| PS_energy_level | 3.63 | 0.73 | 3.22 | 0.69 | 0.577 | 0.277 |
| PS_compassion | 4.21 | 0.63 | 3.93 | 0.66 | 0.434 | 0.212 |
| PS_respectfulness | 4.05 | 0.58 | 4.05 | 0.59 | 0 | 0 |
| PS_trust | 3.37 | 0.80 | 3.15 | 0.63 | 0.306 | 0.151 |
| PS_organization | 3.26 | 0.98 | 3.67 | 0.86 | -0.445 | -0.217 |
| PS_productiveness | 3.05 | 0.85 | 3.48 | 0.71 | -0.549 | -0.265 |
| PS_responsibility | 3.53 | 0.68 | 3.75 | 0.58 | -0.348 | -0.171 |
| PS_anxiety | 3.16 | 0.83 | 2.98 | 0.72 | 0.232 | 0.115 |
| PS_depression | 2.66 | 0.81 | 2.61 | 0.89 | 0.062 | 0.031 |
| PS_emo_volatility | 2.98 | 0.90 | 2.68 | 0.8 | 0.352 | 0.173 |
| PS_aesth_sensitivity | 4.34 | 0.70 | 3.02 | 0.98 | 1.55 | 0.613 |
| PS_intell_curiosity | 4.17 | 0.63 | 3.47 | 0.71 | 1.043 | 0.462 |
| PS_creat_imagination | 4.03 | 0.74 | 3.5 | 0.78 | 0.697 | 0.328 |
| **Need for Cognitive Closure Scale** | | | | | | |
| NCCS | 3.27 | 0.66 | 3.311 | 1.419 | -0.036 | -0.018 |
| **STAXI-2 (State Anger)** | | | | | | |
| S_Ang_dislike | 25.93 | 8.45 | 17.04 | 4.79 | 1.294 | 0.543 |
| S_Ang_F_dislike | 9.90 | 3.71 | 6.16 | 2.45 | 1.19 | 0.511 |
| S_Ang_V_dislike | 9.50 | 3.75 | 5.73 | 1.88 | 1.271 | 0.536 |
| S_Ang_P_dislike | 6.53 | 1.92 | 5.16 | 0.97 | 0.901 | 0.411 |
| S_Ang_neutral | 17.63 | 4.06 | 17.04 | 4.79 | 0.133 | 0.066 |
| S_Ang_F_neutral | 6.37 | 2.05 | 6.16 | 2.45 | 0.093 | 0.046 |
| S_Ang_V_neutral | 5.93 | 1.70 | 5.73 | 1.88 | 0.112 | 0.056 |
| S_Ang_P_neutral | 5.34 | 0.93 | 5.16 | 0.97 | 0.189 | 0.094 |
| S_Ang_rest | 15.83 | 2.86 | 17.04 | 4.79 | -0.307 | -0.152 |
| S_Ang_F_rest | 5.41 | 1.36 | 6.16 | 2.45 | -0.379 | -0.186 |
| S_Ang_V_rest | 5.37 | 1.36 | 5.73 | 1.88 | -0.219 | -0.109 |
| S_Ang_P_rest | 5.05 | 0.63 | 5.16 | 0.97 | -0.134 | -0.067 |

## Participant selection

The online survey ended after 88 people met the following selection criteria regarding the reasons for disliking the pieces: A negative associated memory with the song lead to exclusion, the dislike rating per piece had to be at four or five, the items of high interest (it makes me feel physically uncomfortable, it evoked unpleasant feelings in me, it puts me in a bad mood, it makes me aggressive, I feel stressed) had to have a sum of minimum 19, which represents a minimum mean value of 3.8 on the 5-point scale, the items of low interest (it has no impact on my mood, it makes me sad, I am not moved by it) had to have a sum of maximum 7, which represents a maximum mean value of 2.34 on the 5-point scale. The other items were only used as fillers (and I find it too boring, it has too little emotional expression, I find it musically bad, I find the lyrics bad, I associate negative memories with it). Because less men fit the criteria compared to women, six more men with higher ratings on the ‘low’ dislike category were selected. Because some songs came up more often than others (particularly three songs, Atemlos (Helene Fischer), Last Christmas (Wham), and Despacito (Luis Fonsi)), 20 participants were asked to additionally provide a fourth song.

During the first in-house session, each participant listened to all the songs provided by the other participants. Forty-seven participants finished this session and rated the liking of altogether 197 songs on a 7-point scale from “strongly dislike” (1) to “like very much” (7). Representative excerpts of 30 seconds of each song were presented from Spotify or cut manually. The study was performed in group testing sessions using the software Presentation (Neurobehavioral Systems Inc.), and took max. two hours, including breaks. Once all participants had completed this session, for each disliked song by one participant, a match by another participant who rated the song in the middle of the rating scale was determined, which was a rating of 4 or alternatively 5.

## Procedure of the final session

Participants were informed about the study and then prepared for the recordings of psychophysiological measurements, which included the measurement of the zygomaticus major muscle, corrugator supercilii muscle, and the levator labii superioris alaeque nasi muscle with Ag/AgCl electrodes, respiration rate (RR) with a respiration belt wrapped around the upper rib cage (level with sternum), skin conductance (SCR) with electrodes attached to the index and middle fingers of the left hand, a plethysmograph clip on the ring finger used to measure the blood volume pulse (BVP), and an Ag/AgCl electrode placed on the small finger to measure body temperature. Electrocardiography (ECG) was measured with electrodes placed right below the collarbones and the ground placed on the hip.

For facial EMG, electrodes were placed on the left side of the face. Two electrodes were placed over the eyebrow to measure the activity of the corrugator muscle, while two others were placed on the cheek to measure the zygomaticus muscle activity, and two other on the left side of the nose. The ground electrode was placed in the middle of the forehead. Impedance of facial EMG electrodes was kept below 5 kΩ. Electrodes were connected to the amplifier with a 10s low cutoff for EMG and a DC-cutoff for arousal measures and a 250 Hz high cutoff (to reduce electrical interference). Activity was sampled at a 500 Hz rate.

Participants were comfortably seated in front of a monitor and listened to the music over headphones. Loudness was adjusted to a comfortable level for each participant. Participants were instructed to make the continuous button press with the right hand on a keyboard and change to a computer mouse after the music had ended to fill out the questionnaires. Participants were asked to avoid moving their left hand (with the measures) during recordings.

After filling out the first questionnaires and before the music started, a three minute resting period was recorded after which a cortisol probe was taken. The participant chewed on a cotton stick for one minute (accompanied by a countdown on the screen) and put the cotton back into the Salivette (Saarstedt). This procedure was repeated before each piece. An additional probe was taken at the end of the recordings.

## Analysis

### Preprocessing

Physiological data was cut per piece, i.e., into 3 minutes by 6 pieces. Electrodermal activity was decomposed into tonic and phasic skin conductance components by means of Continuous Decomposition Analysis from the Ledalab toolbox (Benedek & Kaernbach, 2010) with data smoothing using the Gauss method and a width of 16 samples. As we were interested in the event-related changes of skin conductance, we used only the phasic component for further analysis. Electrocardiography (ECG), BVP, respiration, and EMG data were analyzed using the Fieldtrip toolbox (Oostenveld et al., 2011). ECG and BVP signals were bandpass filtered between .8 and 20, and demeaned. QRS complexes from the ECG signal were obtained using *nqrsdetect* in the Fieldtrip toolbox from which interbeat-intervals (IBI intervals) were calculated using *diff* function in MatLab. Respiration data was low pass filter at .6 Hz and demeaned. Maxima peaks in the respiration data were identified and interbreath-intervals (IBrI) were calculated using *diff* function in MatLab. IBI and IBrIs were linearly interpolated at the original sampling rate of 500 Hz to obtain continuous HR and RR. Electromyography data was band-pass filtered between 90 and 130 Hz and Hilbert transformed. The absolute value of the Hilbert transformed signal was then smoothed using the *conv2* function in MatLab. Temperature data was screened to check for artifacts.

## Statistical analysis

Table S2. Results of the factor analysis for the items of music-evoked feelings.

| **Item abbreviation** | **Item** | **Unpleasant feelings** | **Boring/ no effect** | **Sad/ touched** |
| --- | --- | --- | --- | --- |
| *Explained variance* |  | *0.65* | *0.18* | *0.17* |
| e_uncomf_feeling | it triggered unpleasant feelings in me | 0.92 |  |  |
| e_bad_mood | it put me in a bad mood | 0.87 |  |  |
| e_uncomf_body | it was physically uncomfortable | 0.86 |  |  |
| e_funny | I found it funny | -0.45 | -0.30 |  |
| e_boring | I found it boring |  | 0.60 |  |
| e_no_effect | it had no effect on my mood | -0.38 | 0.39 |  |
| e_sad | it made me sad |  |  | 0.72 |
| e_touched | it touched me |  | -0.32 | 0.38 |

# Results

Table S3. Results of the ANOVA. tw = time window, condition = liked or disliked music, rating = continuous pleasantness ratings.

| **SCR** | **NumDF** | **DenDF** | **statistic** | **p.value** |
| --- | --- | --- | --- | --- |
| tw | 3 | 6404.6 | 4.1 | **.006** |
| condition | 1 | 6422.8 | 6.2 | **.013** |
| rating | 3 | 6414.8 | 5.2 | **.001** |
| tw:condition | 3 | 6404.7 | 0 | .99 |
| tw:rating | 9 | 6404.7 | 1.7 | .08 |
| condition:rating | 3 | 6412.9 | 1.3 | .26 |
| tw:condition:rating | 9 | 6404.7 | 0.5 | .884 |
|  |  |  |  |  |
| **HR** | **NumDF** | **DenDF** | **statistic** | **p.value** |
| tw | 3 | 5240.1 | 0.6 | .587 |
| condition | 1 | 5244.5 | 51.9 | **<.001** |
| rating | 3 | 5241.6 | 1.6 | .189 |
| tw:condition | 3 | 5240.1 | 0.3 | .850 |
| tw:rating | 9 | 5240.1 | 0.5 | .895 |
| condition:rating | 3 | 5241.3 | 5.8 | **<.001** |
| tw:condition:rating | 9 | 5240.1 | 0.3 | .982 |
|  |  |  |  |  |
| **Temp** | **NumDF** | **DenDF** | **statistic** | **p.value** |
| tw | 3 | 7090.0 | 0 | .999 |
| condition | 1 | 7090.8 | 100.2 | **<.001** |
| rating | 3 | 7090.4 | 1.2 | .297 |
| tw:condition | 3 | 7090.0 | 0 | .999 |
| tw:rating | 9 | 7090.0 | 0 | 1 |
| condition:rating | 3 | 7090.3 | 3.2 | **.021** |
| tw:condition:rating | 9 | 7090.0 | 0 | 1 |
|  |  |  |  |  |
| **RR** | **NumDF** | **DenDF** | **statistic** | **p.value** |
| tw | 3 | 5873.9 | 10.5 | **<.001** |
| condition | 1 | 5881.8 | 0.2 | .683 |
| rating | 3 | 5878.0 | 1.7 | .164 |
| tw:condition | 3 | 5873.9 | 1.3 | .287 |
| tw:rating | 9 | 5873.9 | 0.5 | .854 |
| condition:rating | 3 | 5877.3 | 1.6 | .192 |
| tw:condition:rating | 9 | 5873.9 | 1.1 | .365 |
|  |  |  |  |  |
| **Levator labii** | **NumDF** | **DenDF** | **statistic** | **p.value** |
| tw | 3 | 7090.0 | 4.9 | **.002** |
| condition | 1 | 7110.1 | 19.0 | **<.001** |
| rating | 3 | 7101.8 | 16.8 | **<.001** |
| tw:condition | 3 | 7090.0 | 1.4 | .243 |
| tw:rating | 9 | 7090.0 | 1.5 | .135 |
| condition:rating | 3 | 7099.1 | 6.0 | **<.001** |
| tw:condition:rating | 9 | 7090.0 | 0.5 | .894 |
|  |  |  |  |  |
| **Corrugator** | **NumDF** | **DenDF** | **statistic** | **p.value** |
| tw | 3 | 7090.4 | 1.6 | .198 |
| condition | 1 | 7113.4 | 4.5 | **.034** |
| rating | 3 | 7104.1 | 16.4 | **<.001** |
| tw:condition | 3 | 7090.4 | 0.3 | .837 |
| tw:rating | 9 | 7090.4 | 2.6 | **.005** |
| condition:rating | 3 | 7101.0 | 0.7 | .538 |
| tw:condition:rating | 9 | 7090.4 | 0.8 | .652 |
|  |  |  |  |  |
| **Zygomaticus** | **NumDF** | **DenDF** | **statistic** | **p.value** |
| tw | 3 | 7090.4 | 1.5 | .218 |
| condition | 1 | 7117.4 | 10.4 | **.001** |
| rating | 3 | 7106.7 | 2.3 | .079 |
| tw:condition | 3 | 7090.4 | 0.3 | .842 |
| tw:rating | 9 | 7090.4 | 0.6 | .796 |
| condition:rating | 3 | 7103.2 | 6.4 | **<.001** |
| tw:condition:rating | 9 | 7090.4 | 0.8 | .652 |

Table S4. Pairwise comparisons following the two-way interaction of rating × condition.

| **Comparison** | **Estimate** | **SE** | **z-ratio** | ***p*** |
| --- | --- | --- | --- | --- |
| **SCR (IA n.s.)** |  |  |  |  |
| **HR** |  |  |  |  |
| neutral pleasant - dislike pleasant | -0.14399 | 0.0511 | -2.815 | .0912 |
| neutral neutral - dislike neutral | -0.08707 | 0.0279 | -3.119 | **.0384** |
| neutral unpleasant - dislike unpleasant | -0.07226 | 0.0242 | -2.991 | .0561 |
| neutral very_unpl - dislike very_unpl | -0.24355 | 0.0367 | -6.644 | **<.0001** |
| **Temperature** |  |  |  |  |
| neutral pleasant - dislike pleasant | -0.10936 | 0.0261 | -4.184 | **.0008** |
| neutral neutral - dislike neutral | -0.10348 | 0.0132 | -7.859 | **<.0001** |
| neutral unpleasant - dislike unpleasant | -0.05680 | 0.0115 | -4.936 | **<.0001** |
| neutral very_unpl - dislike very_unpl | -0.09952 | 0.0179 | -5.570 | **<.0001** |
| **RR (IA n.s.)** |  |  |  |  |
| **Levator labii m.** |  |  |  |  |
| neutral pleasant - dislike pleasant | 0.106423 | 0.0766 | 1.390 | .8624 |
| neutral neutral - dislike neutral | -0.119526 | 0.0386 | -3.098 | **.0410** |
| neutral unpleasant - dislike unpleasant | -0.197097 | 0.0337 | -5.847 | **<.0001** |
| neutral very_unpl - dislike very_unpl | -0.260962 | 0.0524 | -4.984 | **<.0001** |
| **Corrugator supercilii m. (IA n.s.)** |  |  |  |  |
| **Zygomaticus major m.** |  |  |  |  |
| neutral pleasant - dislike pleasant | 0.1625 | 0.0823 | 1.974 | .4996 |
| neutral neutral - dislike neutral | -0.1277 | 0.0415 | -3.079 | **.0433** |
| neutral unpleasant - dislike unpleasant | -0.1356 | 0.0362 | -3.742 | **.0045** |
| neutral very_unpl - dislike very_unpl | -0.2741 | 0.0563 | -4.869 | **<.0001** |

Table S5. Descriptive statistics of the ratings after music listening per condition.

|  | **Condition** | **Liking** | **Pleasantness** | **Valence** | **Pos_act** | **Neg_act** |
| --- | --- | --- | --- | --- | --- | --- |
| Mean | Disliked | 1.33 | 1.53 | 4.87 | 17.4 | 20.8 |
|  | Neutral | 3.62 | 3.76 | 8.44 | 17.2 | 14.1 |
| SD | Disliked | 0.607 | 0.750 | 1.86 | 3.62 | 4.04 |
|  | Neutral | 1.38 | 1.57 | 2.40 | 2.68 | 4.72 |

|  | **Condition** | **S_Ang** | **S_Ang_F** | **S_Ang_V** | **S_Ang_P** |
| --- | --- | --- | --- | --- | --- |
| Mean | Disliked | 25.9 | 9.90 | 9.50 | 6.53 |
|  | Neutral | 17.6 | 6.37 | 5.93 | 5.34 |
| SD | Disliked | 8.56 | 3.76 | 3.80 | 1.95 |
|  | Neutral | 4.11 | 2.07 | 1.72 | 0.938 |

|  | **Condition** | **sad** | **funny** | **touched** | **uncomf_body** | **uncomf_feeling** | **bad_mood** | **no_effect** | **boring** |
| --- | --- | --- | --- | --- | --- | --- | --- | --- | --- |
| Mean | Disliked | 1.94 | 1.46 | 1.88 | 3.97 | 4.46 | 4.22 | 1.60 | 3.34 |
|  | Neutral | 1.59 | 2.46 | 1.99 | 2.14 | 2.44 | 2.24 | 2.31 | 3.02 |
| SD | Disliked | 1.36 | 0.908 | 1.24 | 1.14 | 0.917 | 1.24 | 1.11 | 1.50 |
|  | Neutral | 0.931 | 1.36 | 1.08 | 1.28 | 1.33 | 1.32 | 1.24 | 1.35 |
